# Efficacy of Ultrasound‐Guided Intercostal Nerve Block With Liposomal Bupivacaine for Treating Postherpetic Neuralgia in Adult Patients

**DOI:** 10.1002/kjm2.70277

**Published:** 2026-08-19

**Authors:** Jin Li, Xiang‐Yu Fang

**Affiliations:** ^1^ Department of Anesthesiology Kaihua County People's Hospital Quzhou China; ^2^ Department of Anesthesiology Quzhou People’s Hospital, The Quzhou Affiliated Hospital, Wenzhou Medical University Quzhou China; ^3^ Department of Anesthesiology The First Affiliated Hospital of Wenzhou Medical University Wenzhou China

**Keywords:** herpes zoster, intercostal nerve block, liposomal bupivacaine, pain, postherpetic neuralgia

## Abstract

The study aimed to investigate the efficacy of ultrasound‐guided intercostal nerve block (ICNB) with liposomal bupivacaine (LB) comparing ropivacaine‐dexamethasone (RD) for treating postherpetic neuralgia (PHN) in adult patients. A total of 108 patients were randomized 1:1 to receive ICNB with LB or ICNB with RD. The Visual Analogue Scale scores in the ICNB‐LB were significantly lower than those in the ICNB‐RD at 1 and 2 weeks posttreatment (*p* < 0.001). According to the EuroQol Health‐Related Quality of Life 5‐Dimension, 3‐Level questionnaire (EQ‐5D‐3L), lower proportions of patients reporting problems at 2 weeks posttreatment were observed in the ICNB‐LB regarding the domains of pain/discomfort (*p* = 0.031), usual activities (*p* = 0.027), and symptom of anxiety/depression (*p* = 0.038), versus the ICNB‐RD. The ICNB‐LB had less consumption of pregabalin than the ICNB‐RD at 1 week (*p* = 0.006) and 2 weeks (*p* = 0.013) posttreatment. The rate of pregabalin discontinuation was higher and the proportion of patients who required rescue analgesics was lower in ICNB‐LB versus ICNB‐RD up to Week 2 posttreatment. These results demonstrate LB provides an analgesic superiority through 2 weeks without causing new safety concerns comparing with RD in ultrasound‐guided ICNB for treating adult patients with PHN.

## 1 Introduction

Herpes zoster (HZ), also known as shingles, occurs when the varicella zoster virus (VZV) in dorsal root or cranial nerve ganglia is reactivated and remains a public health issue around the world [1]. Although HZ affect virtually the entire population but its incidence is increasing in those aged 50 years and older due to a natural decline in cell‐mediated immunity to VZV after age 50 [2, 3]. The lifetime risk of HZ is estimated at 25%–30%, rising to 50% among individuals aged 80 years and older [4]. HZ produces painful maculopapular or vesicular rash in a dermatomal distribution, substantially affecting the overall health‐related quality of life (HR‐QoL) of patients and interfering with their activities of daily living [5, 6]. Postherpetic neuralgia (PHN) is the most common complication of HZ and defined as persistent pain following the resolution of the rashes [7]. The incidence rate of PHN in patients with HZ ranges from 5% to 30% depending on definition across 26 countries, with females and older individuals at higher risk [8]. PHN can be treated with topical agents including lidocaine and capsaicin, systemic agents involving opioid analgesics (e.g., tramadol), antidepressants (e.g., amitriptyline), and anticonvulsants (e.g., gabapentin), radiofrequency therapy, or spinal cord electrical stimulation [9, 10, 11].

Nerve blocks, such as paravertebral blocks (PVB) and erector spinae plane (ESP) blocks, with local anesthetics and steroids have been widely used to manage multiple acute postoperative pain and chronic pain including acute HZ and PHN [12]. Compared with PVB block or epidural nerve block, intercostal nerve block (ICNB) is also an effective and safe approach to provide regional anesthesia in the thoracic region and may be encouraged as a promising alternative to relieve HZ‐related pain and prevent PHN [13, 14]. Although not in HZ, ICNB was found to significantly reduce morphine consumption and systemic anesthetic absorption compared to ESP block for patients undergoing uniportal thoracoscopic surgery [15]. The duration of existing local anesthetics for nerve blocks typically lasts only 8–14 h, resulting in breakthrough pain and thus creating a critical need for prolonged local anesthetics [16]. Liposomal bupivacaine (LB) is a multivesicular formulation that slowly releases bupivacaine to extend its efficacy for 72–96 h [17, 18]. Accumulating evidence has shown the use of LB in nerve blocks could extend pain control to 72 h and reduce opioid consumption after thoracoscopic surgery over bupivacaine and ropivacaine [19, 20]. In ICNB, LB was demonstrated with lower opioid consumption and shorter length of stay compared to bupivacaine or ropivacaine [21, 22]. Additionally, LB as a local anesthetic in ultrasound‐guided rectus sheath block has presented clinical advantages, such as superior analgesia and reduced opioid consumption for up to 48 h, over ropivacaine‐dexamethasone (RD) or LB‐dexamethasone regimens [23]. Accordingly, we propose a hypothesis that LB in ICNB could provide superior analgesia with better HR‐QoL for adult patients with PHN over RD. To prove this hypothesis, we performed a randomized trial comparing ultrasound‐guided ICNB using LB versus RD to treat PHN in adult patients, with pain intensity score as a primary efficacy endpoint, HR‐QoL and medication use as secondary endpoints.

## 2 Methods

### 2.1 Trial Design and Participants

This was a single‐center, randomized, comparative study of LB versus RD in ICNB for patients with PHN. Participant recruitment started in January 2025 and ended in December 2025. The study was approved by the Ethics Committees of our center and adhered to the Declaration of Helsinki. Written informed consent was obtained from all patients. Inclusion criteria were (1) persistent neuropathic pain ≥ 3 months after the disappearance of acute herpetic rash; (2) the pain remained moderate to severe [Visual Analogue Scale (VAS) scores (0–10) ≥ 4] at screening and randomization; (3) age ≥ 18 years. The exclusion criteria were: (1) peripheral neuropathy or pain unrelated to PHN; (2) the presence of conditions possibly affecting the assessment of pain; (3) the presence of chronic systemic diseases including but not limited to severe cardiopulmonary, chronic digestive diseases, or neuropsychiatric diseases; (4) severe hematologic, hepatic, or renal abnormalities; (5) known history of allergy to investigational drugs or rescue drug components; (6) women who are pregnant, planning to become pregnant during the study, or are breastfeeding; (7) participated in any other clinical study within 30 days before screening.

### 2.2 Sample Size Calculation

The sample size was calculated based on VAS scores (0–10) and computed with prior‐power analysis using the G*power software (version 3.1.9.2). According to a prior publication of HZ related neuralgia [24], where the mean VAS scores at 4 weeks posttreatment between two group (1.69 vs. 3.18) were significantly different, the required sample size was 45 per group (90 in total) to achieve a power of 90% to detect the difference at a two‐side *α* level of 0.05. To allow for a 20% dropout rate, 54 participants per group (108 in total) were finally included.

### 2.3 Randomization and Blinding

**FIGURE 1:**
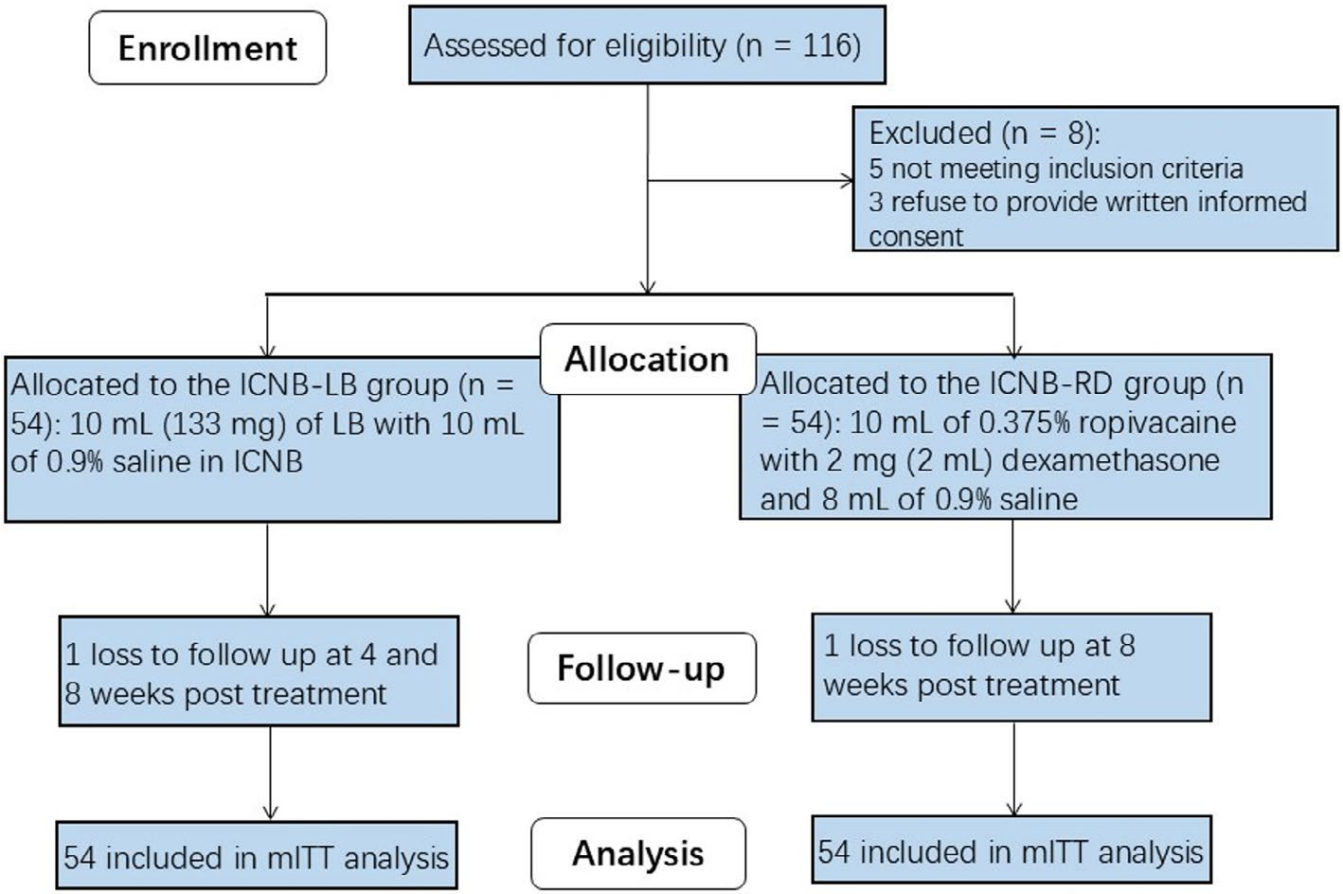
The CONSORT flowchart of patient recruitment. ICNB, intercostal nerve block; LB, liposomal bupivacaine; mITT, modified intent‐to‐treat; RD, ropivacaine‐dexamethasone.

The total sample size was estimated to be 108 participants, and thus 108 patients were randomly allocated in a 1:1 ratio to ICNB‐LB and ICNB‐RD groups via a computer‐generated randomization sequence (SAS statistical package, SAS Institute Inc., Cary, NC, USA) prepared by an independent researcher outside the trial. The generated sequences were then concealed in opaque, sequentially numbered envelopes. A nurse outside the trial opened envelopes in the order of participant enrollment and prepared study medications. The syringes containing local anesthetic were labeled “solution A” or “solution B” to minimize visual clues from solution appearance. Participants, the block provider, outcome assessors, data managers, and statisticians were all unaware of group assignment. The CONSORT flowchart is shown in Figure 1.

### 2.4 Ultrasound‐Guided ICNB Procedures

**FIGURE 2:**
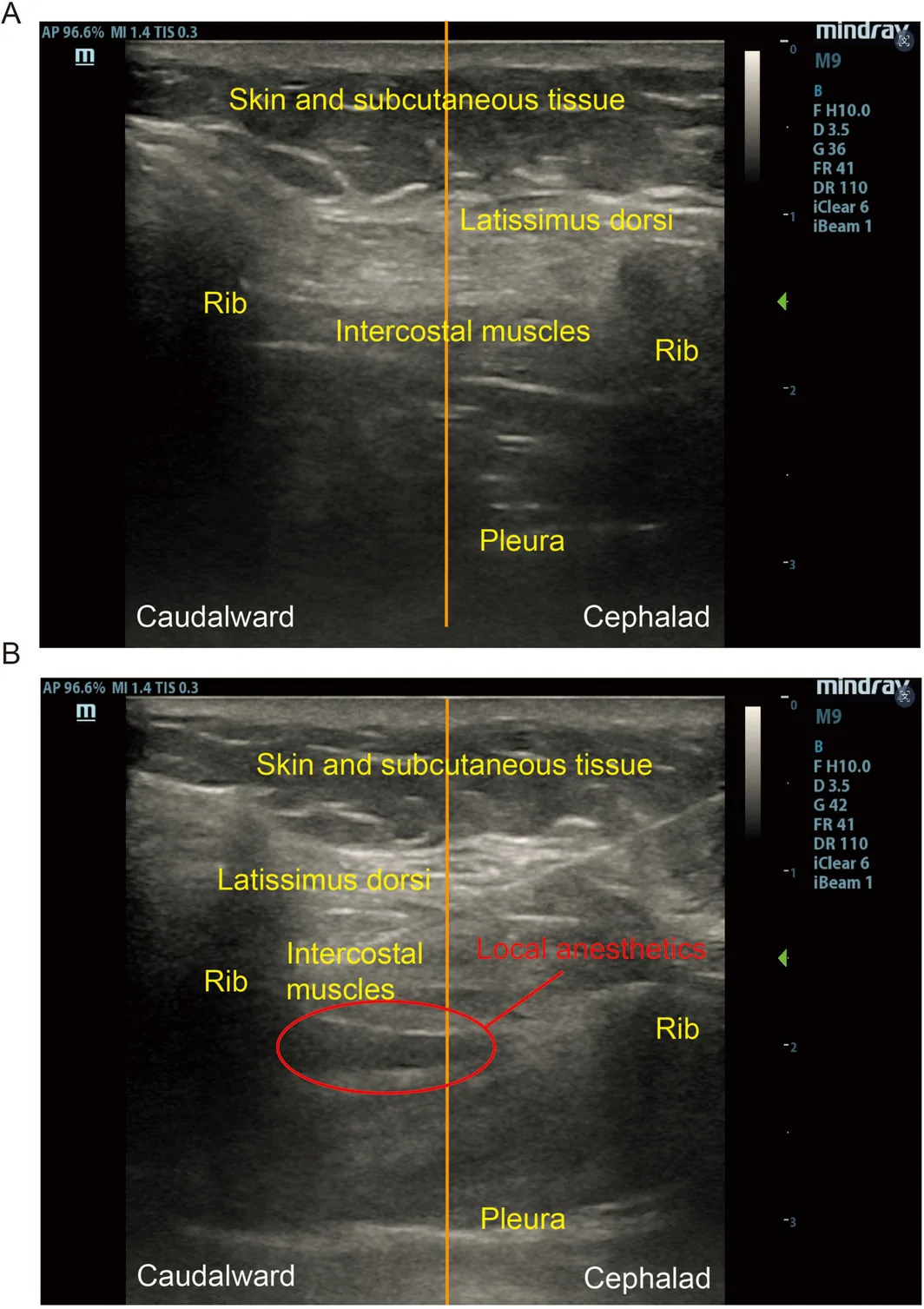
ICNB under ultrasound visualization before (A) and after (B) injection of local anesthetics.

The patients positioned laterally with the painful side upright. All ICNB procedures were performed by the same senior anesthesiologist (Figure 2). A high‐frequency linear ultrasound probe (4–12 MHz frequency) was placed approximately 3–4 cm lateral to the midline of the spine, and its position was adjusted gradually to obtain clear images of the target rib and intercostal space. The targeted intercostal nerve root was identified beneath the color Doppler signal (Mindray UMT‐200, Shenzhen Mindray Bio‐Medical Electronics Co. Ltd., Shenzhen, China) from the corresponding artery. After skin disinfection, a 22‐G nerve puncture needle (Shanghai Ese Medical Technology Co. Ltd., Shanghai, China) was inserted from the caudal to cranial direction in plane under real‐time ultrasound visualization. After negative aspiration, the local anesthetic was injected. ICNB‐LB group: 10 mL (133 mg) of LB (drug name: Aihenping, Jiangsu Hengrui Pharmaceuticals Co Ltd., Jiangsu, China) mixed with 10 mL of 0.9% saline. ICNB‐RD group: 10 mL of 0.375% ropivacaine (drug name: Ruiningshu, Jiangsu Hengrui Pharmaceuticals Co Ltd.) mixed with 2 mg (2 mL) dexamethasone (Tianjin Jinyao Pharmaceutical Co. Ltd., Tianjin, China) and 8 mL of 0.9% saline.

### 2.5 Main Outcomes and Measures

The primary endpoint was resting pain intensity reflected by VAS scores, assessed baseline and at 1 week, 2, 4, and 8 weeks posttreatment. The score of VAS ranges from 0 (“no pain”) to 10 (“the worst pain imaginable”). The secondary endpoints were the HR‐QoL at 2 and 8 weeks posttreatment, the consumption of pregabalin administration at 1 week, 2, 4, and 8 weeks posttreatment, the proportion of patients who were able to discontinue pregabalin medication up to Weeks 2 and 8 posttreatment, and the proportion of patients requiring rescue analgesics up to Weeks 2 and 8 posttreatment. The HR‐QoL assessed by the EuroQol Health‐Related Quality of Life 5‐Dimension, 3‐Level questionnaire (EQ‐5D‐3L) recorded self‐reported problems on each of five dimensions: mobility, self‐care, usual activities, pain/discomfort, and anxiety/depression [25]. Each dimension consists of three levels: no problems, some problems, and extreme problems. All patients were given daily 300 mg pregabalin (150 mg per time, twice daily; Pfizer Manufacturing Deutschland GmbH, Betriebsstatte Freiburg, LYRICA, Germany). If the patients reported VAS ≤ 3, reduced the dose of pregabalin by 75 mg every other day. If the VAS increased to more than 3, the patients were restored to the last controlled pregabalin dosage. Oxycodone and acetaminophen (5:325 mg tablets, 2–3 times/day) were respectively offered as rescue analgesics for patients reporting pain intensity (VAS ≥ 4).

### 2.6 Safety Assessments

Safety assessments were actively performed by an investigator who was blinded to group allocation throughout the 8‐week follow‐up period via in‐person interviews at 1, 2, 4, and 8 weeks after blocks with interim telephone interviews between visits to capture delayed symptoms. All suspected adverse events were documented on standardized safety case report forms and adjudicated by two independent clinicians, including entry point pain, nausea, constipation, pyrexia, dizziness, pruritus, pneumothorax, puncture‐site infection, local hematoma, local anesthetic systemic toxicity symptoms, and neurologic deficit.

### 2.7 Statistical Analysis

When normally distributed (checked via the Shapiro–Wilk test), continuous data were described as a mean value with a standard deviation (SD) or 95% confidence interval (95% CI); otherwise, median with interquartile range (IQR, Q1, Q3) supplemented reporting. Data of mean with SD were analyzed by Student's *t*‐test and data of median with IQR by Mann–Whitney *U* test. Categorical data are summarized in the form of frequencies and proportions, and the Fisher's exact test or *χ* ^2^ test was used as a test of significance. The main efficacy analyses followed a modified intent‐to‐treat (mITT) principle. The full analysis set (FAS) included all randomized patients who received ICNB procedures and had 1 or more postbaseline efficacy evaluations. The VAS scores were analyzed using an analysis of covariance (ANCOVA) model with baseline score as a covariate. The consumption of pregabalin medication was analyzed using repeated measures analysis of variance (ANOVA). If patients in the FAS missed follow‐up measurements, multiple imputation was performed for missing outcome measures using all baseline patient characteristics and outcomes at all time points to generate five imputed data sets. The data set with the highest Cronbach's *α* value was selected for data analysis. The safety set included all patients who received ICNB procedures and had postbaseline safety records. A *p* value (probability that the result is true) less than 0.05 was considered statistically significant. All statistical tests in the study were performed by using the SPSS 27.0 (IBM Corp., Armonk, NY, USA) and the GraphPad prism, version 8.0 (GraphPad, San Diego, CA, USA).

## 3 Results

### 3.1 Baseline Characteristics of Participants

During the study period, among 116 screened patients, 108 were eligible and underwent randomization (54 patients receiving ICNB procedures with LB and 54 receiving ICNB procedures with RD). All 108 patients were treated and had at least one postbaseline efficacy evaluation, and the FAS included 54 patients in each group. There was one patient lost to follow up at both 4 and 8 weeks posttreatment in the ICNB‐LB group and a patient lost to follow up at 8 weeks posttreatment in the ICNB‐RD group. Thus, 53 patients with observed data at 4 and 8 weeks posttreatment in the ICNB‐LB group and 53 patients with observed data at 8 weeks posttreatment in the ICNB‐RD group. Two groups were comparable regarding age, sex, ethnicity, body mass index (BMI), living situation, duration of PHN symptoms, mechanical hyperalgesia/allodynia, previous history of antiviral therapy, and previous medications for acute HZ (Table 1, *p* > 0.05).

**TABLE 1:** Baseline characteristics of participants.

	ICNB‐LB (*n* = 54)	ICNB‐RD (*n* = 54)	*p*
Age, year (mean ± SD)	58.9 ± 9.6	59.4 ± 9.9	0.783
Sex, *n* (%)			0.847
Female	26 (48.2%)	24 (44.4%)	
Male	28 (51.8%)	30 (55.6%)	
Ethnicity, *n* (%)			0.495
Han Chinese	52 (96.3%)	54 (100.0%)	
Other	2 (3.7%)	0 (0.0%)	
BMI, kg/m^2^ (mean ± SD)	22.2 ± 2.4	23.0 ± 2.5	0.109
Living situation, *n* (%)			0.826
Living alone	9 (16.7%)	11 (20.4%)	
Living with partner	36 (66.7%)	33 (61.1%)	
Living with partner and children	9 (16.6%)	10 (18.5)	
Duration of PHN symptoms, month^a^ [median (IQR)]	5.0 (3.3–6.5)	4.7 (3.2–6.3)	0.529
Mechanical hyperalgesia/allodynia, *n* (%)	41 (75.9%)	43 (79.6%)	0.643
Previous antiviral therapy, *n* (%)	45 (83.3%)	49 (90.7%)	0.391
Previous medications, *n* (%)			
NSAID	52 (96.3%)	50 (90.9%)	0.251
Pregabalin	54 (100.0%)	54 (100.0%)	1.000
Oxycodone and acetaminophen	21 (38.9%)	24 (44.4%)	0.558
Tramadol	13 (24.1%)	10 (18.5%)	0.481

Abbreviations: BMI, body mass index; ICNB, intercostal nerve block; LB, liposomal bupivacaine; NSAID, nonsteroidal anti‐inflammatory drug; PHN, postherpetic neuralgia; RD, ropivacaine‐dexamethasone.

^a^ Calculated from the date of onset of neuropathic pain to the date of treatment initiation. Data summarized as mean ± SD are analyzed by unpaired *t‐*test. Data summarized as median (IQR) are analyzed by Mann–Whitney *U* test. Data shown as numbers (percentage) are analyzed by the Fisher's exact test or *χ* ^2^ test.

### 3.2 Primary Efficacy Endpoint

**FIGURE 3:**
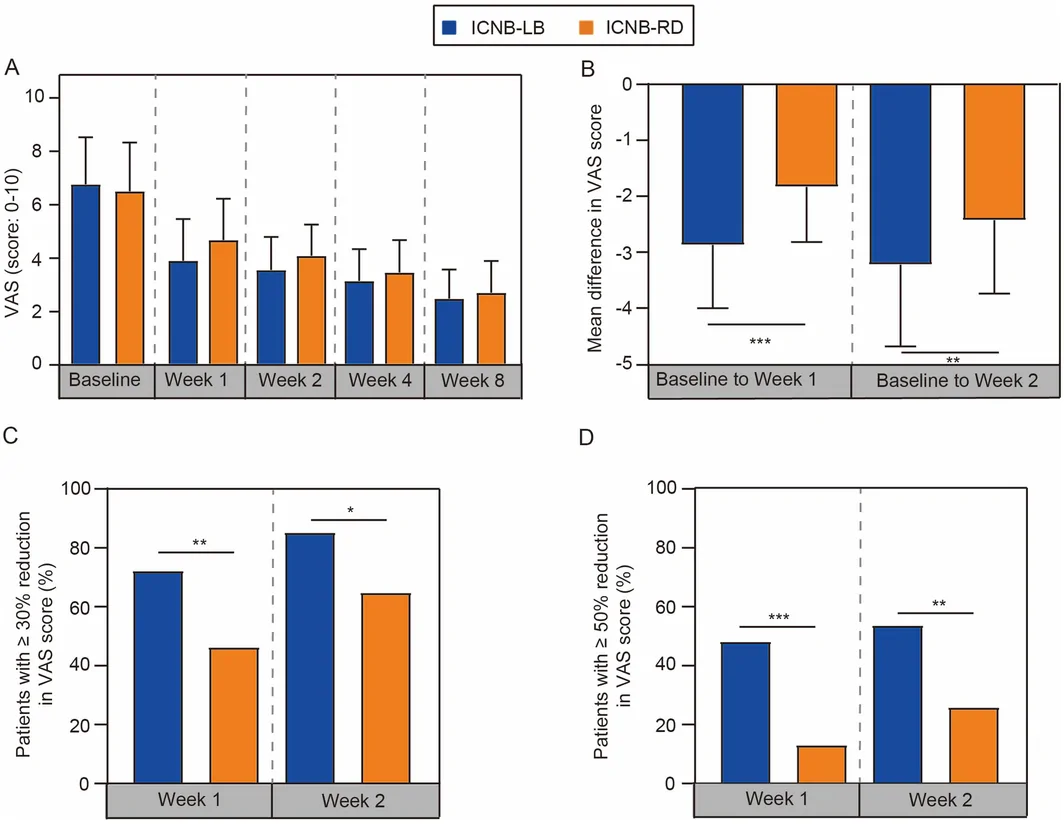
Pain intensity of patients with PHN after ICNB procedures. (A) Graph summary of VAS scores (mean with SD) at baseline and at 1 week, 2, 4, and 8 weeks posttreatment for ICNB‐LB versus ICNB‐RD. (B) Changes of VAS scores (mean with SD) from baseline to 1 and 2 weeks between ICNB‐LB and ICNB‐RD; ANCOVA, **p < 0.01 and ***p < 0.001. (C) The proportion of patients with patients achieving ≥ 30% reduction from baseline to 1 and 2 weeks between ICNB‐LB and ICNB‐RD; Fisher's exact test, *p < 0.05 and **p < 0.01. (D) The proportion of patients with patients achieving ≥ 30% reduction from baseline to 1 and 2 weeks between ICNB‐LB and ICNB‐RD; Fisher's exact test, **p < 0.01 and ***p < 0.001. ICNB, intercostal nerve block; LB, liposomal bupivacaine; RD, ropivacaine‐dexamethasone.

In the FAS, the VAS scores in the ICNB‐LB group were significantly lower than those in the ICNB‐RD group at 1 and 2 weeks after treatment (Figure 3A). At 4 and 8 weeks after treatment, two groups did not differ in the VAS scores. At Week 1, the mean (95% CI) change from baseline in VAS score was −2.9 (−3.2 to −2.6) for ICNB‐LB and −1.8 (−2.1 to −1.6) for ICNB‐RD, with a mean difference (95% CI) of −1.0 (−1.4 to −0.6) versus ICNB‐RD; ANCOVA, *p* < 0.001 (Figure 3B). At Week 2, the mean (95% CI) change from baseline in VAS score was −3.2 (−3.6 to −2.8) for ICNB‐LB and −2.4 (−2.8 to −2.1) for ICNB‐RD, with a mean difference of −0.8 (−1.3 to −0.3); ANCOVA, *p* = 0.005. At Week 1, significantly more patients receiving ICNB‐LB (72.2%) attained at least 30% reduction in VAS scores versus ICNB‐RD (46.3%) (*p* = 0.006; Figure 3C). The proportion of patients with at least 50% reduction in VAS scores was significantly higher with ICNB‐LB (48.2%) vs. ICNB‐RD (13.0%) (*p* < 0.001; Figure 3D). At Week 2, the proportion of patients with at least 30% reduction in VAS scores was significantly higher with ICNB‐LB (85.2%) versus ICNB‐RD (64.8%) (*p* = 0.015), so was the proportion of patients with at least 50% reduction (53.7% vs. 25.9%, *p* = 0.003). No significant difference was noted in 30% or 50% reduction in VAS scores between ICNB‐LB and ICNB‐RD at 4 and 8 weeks (*p* > 0.05).

### 3.3 Secondary Endpoints

**FIGURE 4:**
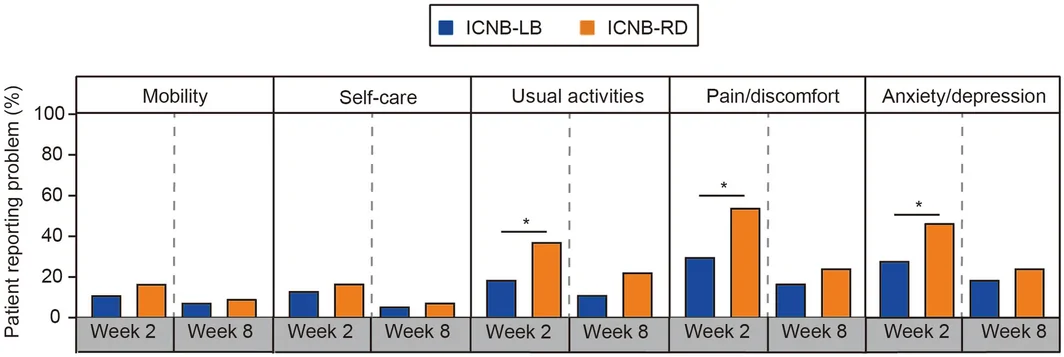
The proportion of patients reporting problems in the domains of the EQ‐5D‐3L between ICNB‐LB and ICNB‐RD; Fisher's exact test, *p < 0.05. ICNB, intercostal nerve block; LB, liposomal bupivacaine; RD, ropivacaine‐dexamethasone.

**FIGURE 5:**
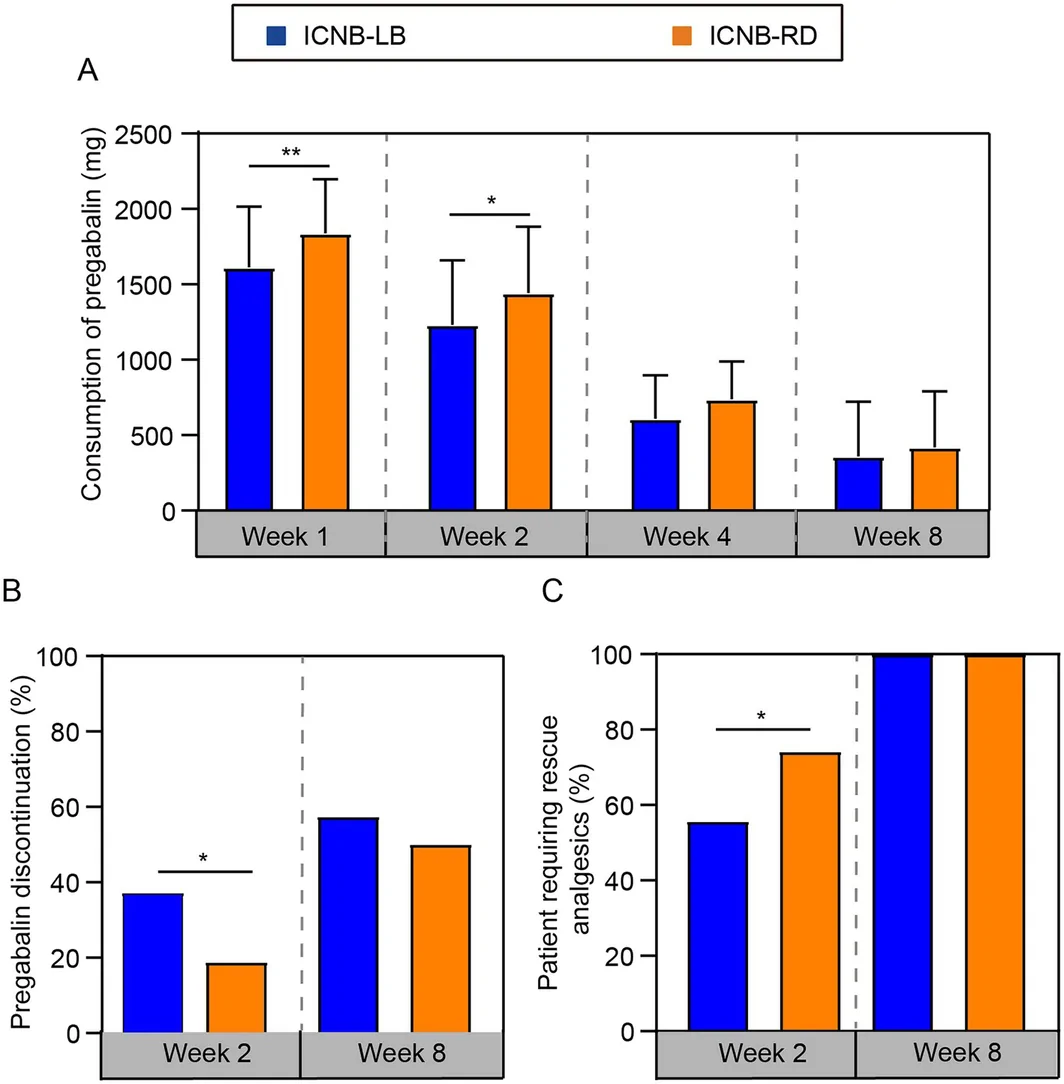
Medication use assessments. (A) The consumption of pregabalin between ICNB‐LB and ICNB‐RD at 1 week, 2, 4, and 8 weeks posttreatment; repeated measures ANOVA, *p < 0.05 and **p < 0.01. (B) The rate of pregabalin discontinuation between ICNB‐LB and ICNB‐RD up to Weeks 2 and 8 posttreatment; Fisher's exact test, *p < 0.05. The proportion of patients who required rescue analgesics between ICNB‐LB and ICNB‐RD up to Weeks 2 and 8 posttreatment; Fisher's exact test, *p < 0.05. ICNB, intercostal nerve block; LB, liposomal bupivacaine; RD, ropivacaine‐dexamethasone.

According to the EQ‐5D‐3L, significant improvements as shown by lower proportions of patients reporting problems at 2 weeks posttreatment were observed in ICNB‐LB regarding the domains of pain/discomfort (29.6% vs. 53.7%, *p* = 0.011), usual activities (18.5% vs. 37.0%, *p* = 0.032), and symptom of anxiety/depression (27.8% vs. 46.3%, *p* = 0.046), vs. ICNB‐RD (Figure 4). Difference between the ICNB‐LB and ICNB‐RD were not significant at 8 weeks posttreatment (*p* > 0.05). The repeated measures ANOVA suggested less consumption of pregabalin in the ICNB‐LB group than the ICNB‐RD group at 1 week (mean difference = −225 mg; 95% CI: −402 to −48; *p* = 0.006) and 2 weeks (mean difference = −209 mg; 95% CI: −386 to −32; *p* = 0.013) posttreatment (Figure 5A). The rate of pregabalin discontinuation was significantly higher in the ICNB‐LB than in the ICNB‐RD up to Week 2 posttreatment (37.0% vs. 18.5%, *p* = 0.032) (Figure 5B). Difference of pregabalin discontinuation between the ICNB‐LB and ICNB‐RD were not significant up to Week 8 posttreatment (57.4% vs. 50.0%). The proportion of patients who required rescue analgesics was significantly lower in the ICNB‐LB than in the ICNB‐RD up to Week 2 posttreatment (55.6% vs. 74.1%, *p* = 0.044) (Figure 5C). Difference between the ICNB‐LB and ICNB‐RD were not significant up to Week 8 posttreatment (100% vs. 100%, *p* > 0.05).

### 3.4 Safety Evaluations

Within the follow‐up period of 8 weeks, there were no serious drug‐ or block‐related adverse events in the present study. No significant difference was noted in patients experiencing entry point pain or dizziness within postprocedural 15 min between the ICNB‐LB and ICNB‐RD (*p* > 0.05, Table 2).

**TABLE 2:** The incidence of adverse events between the ICNB‐LB and ICNB‐RD.

	ICNB‐LB (*n* = 54)	ICNB‐RD (*n* = 54)	*p*
Adverse events, *n* (%)	19 (35.2%)	16 (29.6%)	0.681
Entry point pain	13 (24.1%)	11 (20.4%)	
Dizziness	6 (11.1%)	5 (9.3%)	
Nausea	0 (0.0%)	0 (0.0%)	
Constipation	0 (0.0%)	0 (0.0%)	
Pyrexia	0 (0.0%)	0 (0.0%)	
Pruritus	0 (0.0%)	0 (0.0%)	
Pneumothorax	0 (0.0%)	0 (0.0%)	
Puncture‐site infection	0 (0.0%)	0 (0.0%)	
Local hematoma	0 (0.0%)	0 (0.0%)	
Local anesthetic systemic toxicity symptoms	0 (0.0%)	0 (0.0%)	
Neurologic deficit	0 (0.0%)	0 (0.0%)	

*Note:* Data shown as numbers (percentage) are analyzed by the Fisher's exact test or *χ* ^2^ test.

Abbreviations: ICNB, intercostal nerve block; LB, liposomal bupivacaine; RD, ropivacaine‐dexamethasone.

## 4 Discussion

The results of this randomized trial proved the primary hypothesis of significant pain relief at 1 and 2 weeks after intervention and the secondary hypotheses of lower proportions of patients reporting problems in HR‐QoL related domains and requiring rescue analgesics at 2 weeks posttreatment, provided by LB in ICNB compared to RD. Safey profiles were comparable with no serious adverse events.

When the nerves, roots, and ganglion were damaged due to reactivation of VZV, their ability to inhibit nociceptive pain signals was lost. This reduced the threshold for nociceptive pain activation and resulted in spontaneous ectopic discharges, thus producing hyperalgesia and allodynia [26]. Besides, the C fibers became sensitized due to small fiber deafferentation from damage and their potentials were evoked, thus leading to spontaneous pain and allodynia mediated by peripheral nervous system [27]. Overall, the mechanism of PHN may be associated with constant pain arising from a marked loss of nociceptive afferents as well as paroxysmal pain resulting from Abeta‐fiber demyelination [28]. Although the PVB is one of the most commonly applied peripheral nerve blocks to reduce acute HZ‐related pain, this block may cause the most common development of inadvertent vascular puncture, followed by hematoma, hypotension, pleural puncture, and pneumothorax [29]. According to the anatomy, the space between the adjacent ribs is usually wider and more superficial than that between the two thoracic transverse processes, which allows a less steep needle angle trajectory to achieve superior real‐time ultrasound visualization of key anatomical landmarks compared to PVB block [30]. Importantly, in cases receiving ICNB procedures, shorter procedure time was observed, which demonstrated that ICNB was an easier and time‐efficient approach [13]. In chronic neuropathic pain (such as PHN), the nervous system undergoes plasticity changes [31]. After peripheral nerve injury, the damaged area will continue to produce ectopic discharges, and these abnormal incoming signals continuously affect the central nervous system (spinal cord and brain), leading to abnormally increased sensitivity of central neurons (central sensitization) [32]. The potential effect of a single peripheral nerve block to improve PHN may be explained by the following hypotheses. Local anesthetics can reversibly inhibit the generation and transmission of nerve impulses by blocking the sodium ion channels of neurons, thereby temporarily eliminating abnormal incoming signals from peripheral lesions [33]. During this stage, the central nervous system is able to escape from abnormal stimuli, which provides an opportunity for central neurons to recover from pathological hypersensitivity, eliminate or alleviate the function of established pathological pain circuits, and thus producing therapeutic effects beyond the analgesic time of the drug itself.

The analgesic benefits of conventional local anesthetics, even with adjuvants such as dexamethasone and dexmedetomidine, are often limited to less than 24 h [34]. LB in ICNB yielded decreased pain intensity scores, which may be attributed to extended perineural residence and sustained release of bupivacaine from multiple, nonconcentric lipid bi‐layers [18]. Extended local anesthetic availability due to prolonged release of LB can prevent or reduce the transmission of nociceptive signals to the central nervous system, thereby reducing central sensitization to pian. The pharmacokinetics of LB following multilevel ICNB demonstrate a significant delay in systemic absorption of the drug [35]. In accordance with what was expected, we observed decreased pain intensity scores at 1 and 2 weeks after ICNB procedures in LB versus RD. Yan et al. [36] demonstrate ICNB with LB was associated with reduced incidence of chronic postsurgical pain (a numerical rating scale pain score ≥ 1) after video‐assisted thoracoscopic surgery compared with ropivacaine, which suggesting the role of LB in preventing chronic pain. The presence and severity of neuropathic pain are related to greater impairments in several important HR‐QoL domains [37]. Significant pain relief offered by LB over RD until 2 weeks after ICNB procedures may allow less impaired HR‐QoL of patients with PHN.

This study has several limitations. One limitation concerns the single‐center nature and modest sample size of Chinese patients, which makes whether these findings are applicable to the global population to be investigated. Many of outcome data were collected based on patient recall, which may introduce bias. However, this recall bias would apply similarly in both cohorts. Besides, there was lack of detailed control for concomitant analgesic use in the ICNB‐LB group, creating a future need of use of LB with dexamethasone in ICNB. Although we provided previous medications of patients at baseline, their consumption of pregabalin and tramadol was absent at baseline. Further study populations with detailed consumption of medications are warranted to better validate the sparing effect of comedication after ICNB.

In conclusion, our results demonstrate LB is safe and effective for ICNB, providing an analgesic superiority with a better HR‐QoL through 2 weeks for adult patients with PHN. No new safety concerns emerged with LB. We recommend further studies to compare other adjuvant medications that can potentially increase the efficacy of ICNB with LB in order to achieve more persistent analgesia. Considering relatively higher costs of LB compared to other local anesthetics, its cost efficiency should be further investigated to enhance its availability and feasibility in resource‐limited settings.

## Funding

The study was supported by the Clinical Research Fund Project of Pain Physicians Branch of Zhejiang Medical Doctor Association in 2024: YS2024‐50‐007.

## Conflicts of Interest

The authors declare no conflicts of interest.

## Data Availability

The data that support the findings of this study are available from the corresponding author upon reasonable request.
